# Influence networks based on coexpression improve drug target discovery for the development of novel cancer therapeutics

**DOI:** 10.1186/1752-0509-8-12

**Published:** 2014-02-05

**Authors:** Nadia M Penrod, Jason H Moore

**Affiliations:** 1 Department of Pharmacology and Toxicology, Geisel School of Medicine at Dartmouth College, HB7937 One Medical Center Dr., Lebanon, NH 03766, USA; 2 Department of Genetics, Geisel School of Medicine at Dartmouth College, HB7937 One Medical Center Dr., Lebanon, NH 03766, USA; 3 Institute for Quantitative Biomedical Sciences, Geisel School of Medicine at Dartmouth College, HB7937 One Medical Center Dr., Lebanon, NH 03766, USA

**Keywords:** Breast cancer, Coexpression networks, Drug target discovery, Letrozole, Tumor adaptation

## Abstract

**Background:**

The demand for novel molecularly targeted drugs will continue to rise as we move forward toward the goal of personalizing cancer treatment to the molecular signature of individual tumors. However, the identification of targets and combinations of targets that can be safely and effectively modulated is one of the greatest challenges facing the drug discovery process. A promising approach is to use biological networks to prioritize targets based on their relative positions to one another, a property that affects their ability to maintain network integrity and propagate information-flow. Here, we introduce influence networks and demonstrate how they can be used to generate influence scores as a network-based metric to rank genes as potential drug targets.

**Results:**

We use this approach to prioritize genes as drug target candidates in a set of ER ^+^ breast tumor samples collected during the course of neoadjuvant treatment with the aromatase inhibitor letrozole. We show that influential genes, those with high influence scores, tend to be essential and include a higher proportion of essential genes than those prioritized based on their position (i.e. hubs or bottlenecks) within the same network. Additionally, we show that influential genes represent novel biologically relevant drug targets for the treatment of ER ^+^ breast cancers. Moreover, we demonstrate that gene influence differs between untreated tumors and residual tumors that have adapted to drug treatment. In this way, influence scores capture the context-dependent functions of genes and present the opportunity to design combination treatment strategies that take advantage of the tumor adaptation process.

**Conclusions:**

Influence networks efficiently find essential genes as promising drug targets and combinations of targets to inform the development of molecularly targeted drugs and their use.

## Background

As we continue the pursuit of personalized medicine in cancer, where treatments are matched to the molecular signature of individual tumors, the demand for new drugs will continue to rise. Oncology drugs have historically had high attrition rates in late-phase clinical trials due to safety and toxicity concerns or a lack of efficacy [1,2]. The advent of molecularly targeted drugs has improved attrition rates bringing more drugs to market [3], but the efficacy of these drugs is often short-lived as tumors adapt to become resistant to their effects. To support the development of novel drugs we need efficient ways to discover better druggable targets to selectively kill cancerous cells and better combinations of targets to prevent resistance.

Network biology is an attractive platform for the discovery of novel drug targets because it captures both the detail of individual molecular interactions and a global snapshot of how these interactions fit together to create a functional system. In this way, the network becomes a map that can be used to strategically find points of intervention, based on the context-dependent relationships of the potential target, and that can be modulated to tweak the biological function of the entire system. To identify targets for the treatment of cancer, the network can be used to identify points of vulnerability that when targeted will lead to the loss of viability.

Measures of node centrality, or how well a node is embedded within the network, are one possible way to identify such targets. Nodes that have many links, called hubs, and those that lie in well-traveled paths, called bottlenecks, are integral for maintaining network integrity and paths of information-flow [4]. Genes or gene-products that occupy these positions within a network tend to be essential [4,5] which means they are required for viability, and thus they become promising drug targets for the treatment of cancer.

**Figure 1:**
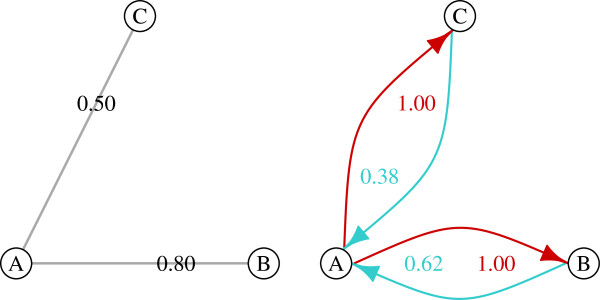
**Generating influence networks.** Influence networks are directed weighted networks (right) generated from undirected weighted networks (left). Illustrated here is an example of a coexpression network and its influence network. Influence is calculated as the proportion of total edge weights a given node shares with each of its neighboring nodes (see Methods). Here, red links show the influence of node A on nodes B and C, which are completely influenced by node A because they do not have other neighbors to influence them. Blue links show the influence of nodes B and C on node A. Node B has a greater influence on node A than node C because the weight of the edge linking B to A is greater than the weight of the edge linking C to A. The cumulative influence of each node is calculated by summing the values of all outgoing edge weights from a given node.

Here we introduce a network-based method for drug target discovery called influence networks. Influence networks convert weighted undirected networks into weighted bidirectional networks where pairs of nodes are connected by two links, one weighted to represent the influence a given node has on its partner and the other weighted to represent the influence the partner has on the given node (Figure 1). From these networks we calculate a cumulative influence score as a measure of the importance of each gene within the network. Using these scores we rank the genes as potential drug targets from most influential to least influential.

We apply this method to coexpression networks generated from transcriptional profiles of ER ^+^ breast tumors before, during, and after a course of neoadjuvant treatment with the drug letrozole. We show that highly influential nodes tend to be essential, influential nodes are more often essential than hubs or bottlenecks, and influential genes make promising drug target candidates.

## Methods

### Gene expression data

Transcriptional profiles were generated from core biopsies extracted from ER ^+^ breast tumors during the course of neoadjuvant treatment with the aromatase inhibitor letrozole [6,7]. Tumors were sampled before treatment (n = 58), following 14 days (n = 58), and following 90 days (n = 60) on drug. Inclusion criteria required samples to contain at least 20% malignant tissue. RNA was extracted from each sample, amplified, and hybridized to Affymetrix HG-U133A GeneChip arrays. The data can be accessed through the Gene Expression Omnibus (GEO) database (GSE20181).

### Gene expression data processing

The raw probe intensity (CEL) files were downloaded from GEO. We processed the data with a custom chip definition file (CDF) to assign the most recent probe annotations and to exclude promiscuous probes from further analysis [8]. We used the R implementation of the robust multi-array average (RMA) algorithm to background correct, normalize, and summarize the data [9].

### Differential expression analysis

To select the subset of genes that are affected by letrozole treatment we calculated differential expression between genes in the tumor samples collected before treatment and those collected following 90 days of treatment. Differential expression analysis was done using the Linear Models for Microarray Data (limma) method [10] implemented in the limma package in R. This method models the affect each treatment condition has on the expression level of each gene. We chose this method because of its demonstrated robust performance across a variety of sample sizes and noise levels [11]. We correct for multiple hypothesis testing by setting a false discovery rate (FDR) at 5% and genes below this value were considered differentially expressed at a statistically significant level. We used the set of differentially expressed genes to perform coexpression analysis.

### Coexpression networks

To generate sets of coexpressed gene pairs we calculate the 1st-order Spearman’s correlation coefficient [12] among all pairs of differentially expressed genes. Spearman’s correlation allows us to identify both linear and nonlinear relationships and the 1st-order correlation, or partial correlation, ensures we are only identifying direct gene-gene relationships by removing associations that appear due to common regulators. We generated three sets of coexpressed gene pairs: those that occur in the untreated tumors, those that occur following 14 days of letrozole treatment, and those that occur following 90 days of letrozole treatment.

Based on simulations carried out by de la Fuente et al. [12], we chose a significance threshold of *α*=0.01. We validated this threshold through permutation testing where the expression levels of each gene are randomized, within each time point, across samples. From the randomized data we calculated coexpression as described and counted the number of gene-gene relationships that meet our significance threshold. This process was repeated 1000 times, and the counts were used to generate null distributions for each time-point. The observed number of coexpression relationships, for each of the three time-points, fall to the right, outside of the matched null distribution.

We used the igraph package in R [13] to convert the lists of pairwise coexpression relationships into undirected, weighted coexpression networks where nodes represent genes and edges connect pairs of genes that have a statistically significant partial correlation relationship. Edges were weighted to reflect the partial correlation coefficient shared between the pair of connected nodes. We generated three coexpression networks, one for the untreated tumor samples, one for those that have been treated with letrozole for 14 days, and one for those that have been treated with letrozole for 90 days.

### Influence networks

To generate influence networks from the weighted, undirected coexpression networks we applied the methods developed by Hangal et al. [14] (Figure 1) to get bidirectional, weighted edges by calculating the influence each node has on its neighbors as follows:

(1) $$\text{Influence}(A,B)=\frac{\text{pcor}(B,A)}{\sum_{X}\text{pcor}(B,X)}$$

where Influence(A,B) is the influence of node A on node B given the strength of node B’s partial correlation with node A in proportion to the strength of node B’s partial correlation with all other nodes denoted by X.

From the influence networks, we calculate a cumulative influence score for each node as the sum of its influences on all other nodes as follows:

(2) $$\text{Influence}(A)=\underset{X}{\sum }\text{Influence}(A,X)$$

where Influence(A) is the influence score of node A.

### Other network measures of influence

In general, nodes with high centrality scores tend to be essential in complex networks, including those derived from biological systems [4,5]. The two most often cited measures of centrality are degree centrality and betweenness centrality. Degree centrality is calculated by counting the number of edges of a given node. Nodes with high degree centrality are well-connected and referred to as hubs. Betweenness centrality is calculated as the number of shortest paths between all pairs of nodes that cross a given node. Nodes with high betweenness centrality control the rate of information flow and are referred to as bottlenecks.

Here we calculated the degree and betweenness centralities of the influence networks using the igraph package in R [13]. We calculated degree for outgoing edges only because every edge is bidirectional. We calculated betweenness with respect to the weighted and directed edges.

### Gene essentiality

To identify genes that are essential for breast cancer cells to proliferation and survive in culture, we downloaded genome-wide pooled shRNA screen data across 29 breast cancer cell-lines from the COLT-Cancer database [15]. Each cell-line was screened in triplicate and a Gene Activity Ranking Profile (GARP) score, with an associated p-value, was developed by Marcotte et al. [16] to reflect the essentiality of a given gene within a given cell-line. Because tumors are heterogeneous and we do not know which cell-lines best model the patient data used in our analysis, we define essentiality as having a significant GARP score (p ≤ 0.05) in at least one of the 29 breast cancer cell-lines.

We calculated the proportion of influential genes in the untreated tumors that are also essential. For comparisons between measures of nodal importance, we calculated the proportion of genes that are essential among genes ranking in the top 25 for influence score, degree, or betweenness centrality. We chose this number based on our prior characterization of these networks where we classified genes as hubs or bottlenecks if their degree or betweenness centrality scores, respectively, were statistical outliers [17]. For consistency, we choose the top influential genes as those that are statistical outliers by their influence scores. The numbers of genes comprising each of the categories across networks varies so we choose to compare the greatest common number across categories which is 25.

## Results

### Influence networks

We first generated undirected weighted coexpression networks from the set of genes that are differentially expressed in ER ^+^ breast tumor samples following neoadjuvant treatment with the drug letrozole. We focus on the set of differentially expressed genes to capture adaptation to letrozole treatment at the expression level. To capture dynamic coexpression changes throughout the course of treatment, we generated three networks: one representing the untreated tumors, one representing early changes following 14 days on letrozole, and one representing late changes following 90 days on letrozole. In these networks, nodes represent genes and two genes are connected by an edge if their expression levels are correlated by a statistically significant 1st-order Spearman’s correlation coefficient. Edges were weighted by taking the absolute value of the correlation coefficients that connect coexpressed pairs of genes.

Coexpression networks based on correlation are undirected because correlation is a symmetric measure of dependence. In other words, when the expression levels of two genes, A and B, are correlated the interpretation is that the level of gene A depends on the level of gene B to an equal extent that the level of gene B depends on the level of gene A. However, we know that the relationships between genes are actually asymmetric. For example, if gene A is a transcription factor that regulates the expression of gene B, the influence of A on B is usually greater than the influence of B on A. This distinction becomes critical when identifying influential genes as potential drug targets.

To capture asymmetrical relationships between genes, we converted our undirected weighted coexpression networks into directed weighted influence networks using a method developed by Hangal et al. [14] (Figure 1). This produces networks in which each correlated gene pair is connected by two edges of opposite direction where one edge reflects the influence of a given node on its partner and the other edge reflects the amount a given node is influenced by its partner. We sum the weights of the outgoing edges for each node to calculate a cumulative influence score for each gene.

### Influence and essentiality

**Figure 2:**
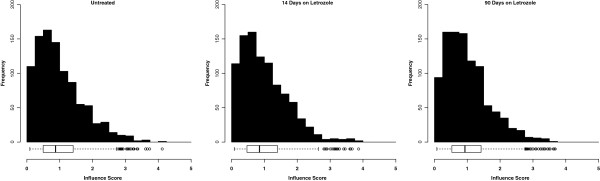
**Influence score distributions.** Distributions of influence scores within coexpression-based influence networks representing ER ^+^ breast tumors before, during, and after neoadjuvant letrozole treatment. These heavy-tailed distributions show that most genes have limited influence while a few genes are highly influential. Boxplots show the spread of the influence scores including the median value and statistical outliers under each treatment condition.

Influence is not a uniformly distributed property across nodes. Most genes have limited influence. A few genes are highly influential. The distributions of influence scores consistently show this pattern at each of the three time-points (Figure 2).

**Figure 3:**
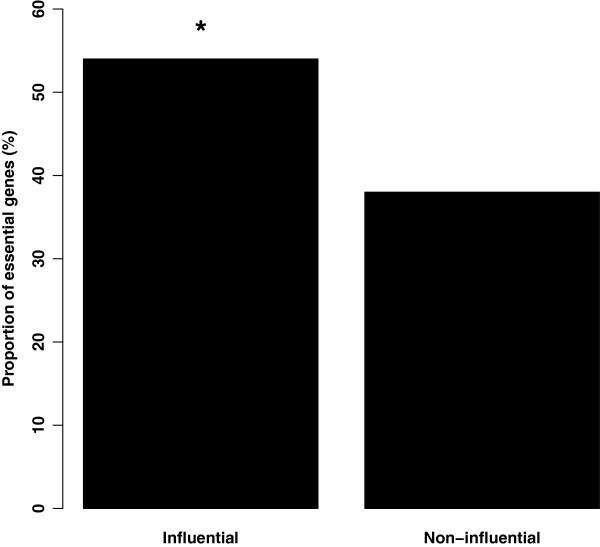
**Influential genes are enriched for essential genes.** Influential genes, identified with the coexpression based influence network representing untreated ER ^+^ breast tumors, are enriched for essential genes in contrast to noninfluential genes within the same network (p <.001). p-value calculated using the cumulative binomial distribution.

We hypothesized that highly influential genes will be critical for maintaining the integrity of the network and thus essential to the tumors. To test this, we calculated the proportion of influential genes, selected as statistical outliers by influence score, in the untreated tumor network that are identified as essential genes in an independent shRNA screen of breast cancer cell-lines in the absence of drug treatment [15]. We found that the set of influential genes are significantly enriched for essential genes (*p*<.001, cumulative binomial distribution) (Figure 3). We show this enrichment holds for a range of thresholds (Additional file 1: Figure S1).

### Influence and centrality

It has been shown that nodes that have high degree and/or betweenness centralities are frequently associated with essentiality [4,5]. To determine if the influence metric provides different information than measures of centrality, we compare the degree and betweenness for each gene in the untreated tumor network with its corresponding influence score.

**Figure 4:**
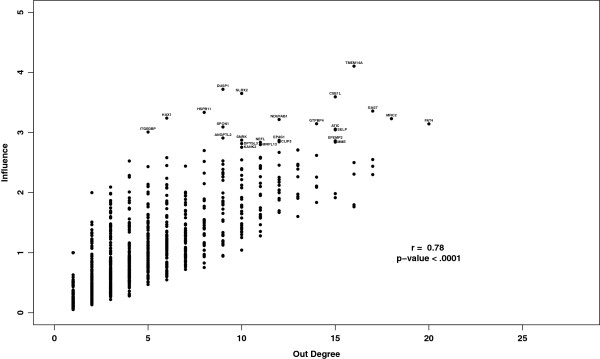
**Influence and degree centrality.** The influence scores of genes within the coexpression based influence network of untreated ER ^+^ breast tumors are correlated with their degree. Although influential nodes do not necessarily have high degree and nodes of high degree are not necessarily influential. Labels correspond to the most influential genes presented in Table 1.

We found that the degree of a gene and its influence are highly correlated (r = 0.78, *p*<.001) (Figure 4) which is consistent with the quantitative definition of influence. However, the most influential genes are not consistently found among those with the highest degree, so choosing candidates based on a degree threshold would exclude many genes of high influence.

**Figure 5:**
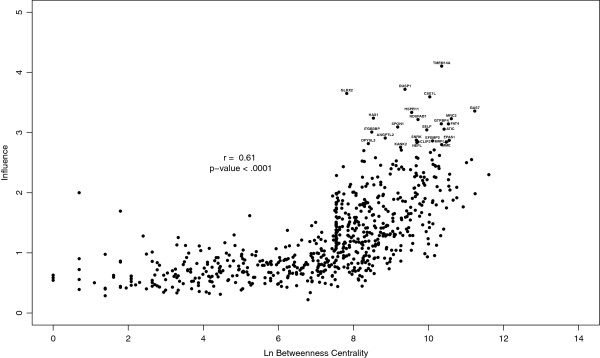
**Influence and betweenness centrality.** The influence scores of genes within the coexpression based influence network of untreated ER ^+^ breast tumors are correlated with their betweenness centrality. Although influential nodes do not necessarily have high betweenness and nodes of high betweenness are not necessarily influential. Labels correspond to the most influential genes presented in Table 1.

The betweenness of a gene and its influence are also correlated (r = 0.61, *p*<.001) (Figure 5). The most influential genes tend to fall among those with high betweenness scores but there is a lot of variability in influence at these values of betweenness. Therefore, choosing candidates based on a betweenness threshold would include many genes that have little influence.

We also calculated the proportion of essential genes among those with the highest influence, degree, or betweenness measures. We find a greater proportion of essential genes among the influential nodes than among hubs (*p*<.001, Fisher’s exact test) or bottlenecks (p <.001, Fisher’s exact test). We show this trends is maintained across a range of thresholds (Additional file 2: Figure S2).

### Influence to identify druggable targets

Having shown that influence identifies essential genes, we selected the most influential genes within each network for assessment as potential drug targets (Table 1). We are looking for targets that will synergize with letrozole treatment, and by using sequential biopsy samples during the course of treatment, we have the unique opportunity to identify targets in the untreated tumors and in the residual tumors after they have been rewired in an adaptive response to 14 days and 90 days of drug treatment. Targeting these induced-essential genes additively with letrozole as they become essential has the potential to stave off resistance.

**Table 1:** Most influential genes

	**Untreated**		**14 Days Letrozole**		**90 Days Letrozole**	
**Rank**	**Entrez ID**	**Gene symbol**	**Entrez ID**	**Gene symbol**	**Entrez ID**	**Gene symbol**
1	28978	**TMEM14A**	79776	**ZFHX4**	1727	**CYB5R3**
2	1843	DUSP1	4701	**NDUFA7**	7083	TK1
3	51022	GLRX2	715	**C1R**	1292	**COL6A2**
4	1434	**CSE1L**	79818	ZNF552	857	**CAV1**
5	8522	**GAS7**	4046	**LSP1**	5437	POLR2H
6	51668	HSPB11	1727	**CYB5R3**	9833	MELK
7	10456	HAX1	357	SHROOM2	54861	**SNRK**
8	9902	**MRC2**	283298	**OLFML1**	4830	**NME1**
9	4706	NDUFAB1	23179	RGL1	231	AKR1B1
10	23560	**GTPBP4**	1809	**DPYSL3**	10418	SPON1
11	79633	**FAT4**	857	**CAV1**	2350	FOLR2
12	10418	SPON1	274	BIN1	10979	**FERMT2**
13	471	**ATIC**	79833	GEMIN6	11096	**ADAMTS5**
14	6403	**SELP**	11222	MRPL3	4638	**MYLK**
15	23421	ITGB3BP	23244	PDS5A	9448	MAP4K4
16	23452	ANGPTL2	6241	RRM2	54922	RASIP1
17	54861	SNRK	1289	**COL5A1**	6934	TCF7L2
18	2034	EPAS1	8434	**RECK**	4072	**EPCAM**
19	30008	**EFEMP2**	429	ASCL1	4701	NDUFA7
20	25999	CLIP3	3426	CFI	404672	GTF2H5
21	4311	**MME**	332	BIRC5	6241	RRM2
22	4747	NEFL	2	**A2M**	2192	**FBLN1**
23	1809	DPYSL3	10730	YME1L1	25959	**KANK2**
24	28998	MRPL13	11130	ZWINT	55651	NHP2
25	25959	KANK2	1292	**COL6A2**	5050	PAFAH1B3

Lists of the top 25 of most influential genes in influence networks based on coexpression among genes in ER ^+^ breast tumors before, during, and after a course of neoadjuvant treatment with the drug letrozole. Bold font indicates genes that are also classified as hub-bottleneck genes.

**Figure 6:**
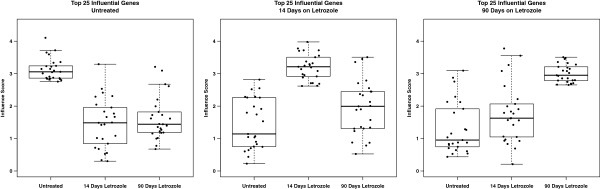
**Influence scores among top candidates across networks.** The influence scores of the most influential genes (Table 1) within a time point are shown across each of the three treatment conditions illustrating that influence is a condition-dependent property. For each comparison, the influence scores among the top genes at a given time-point are statistically significantly higher than their influence scores at the other time-points (p <.001, Student’s t-test).

We compare the influence scores among the most influential genes under each treatment condition with their corresponding scores at the two other treatment conditions (Figure 6). As expected, in all cases, the influence scores among the top genes at a given time-point are statistically significantly higher than their influence scores at the other time-points (p <.001, Student’s t-test). We also show that changes in influence scores among the genes within these sets are larger than would be expected by random chance (Additional file 3: Figure S3).

The genes with the highest influence are the transmembrane protein TMEM14A in the untreated tumor samples, the zinc-finger transcription factor ZFHX4 following 14 days of letrozole treatment, and the redox protein CYB5R3 following 90 days of letrozole treatment. Each of these genes have been associated with cancer although their functional roles are still under investigation making them novel potential targets.

TMEM14A has been shown to play a functional role in the suppression of apoptosis by inhibiting the pro-apoptotic protein Bax and by regulating mitochondrial membrane potential [18]. TMEM14A may also play a role in the regulation of planar cell polarity by trafficking planar cell polarity proteins to the membrane including VANGL2 [19], a protein that promotes migration and invasion in human cancer cells [20].

ZFHX4 is a gene of unknown function. In beef cattle it has been associated with the regulation of puberty through predicted participation in protein-protein interactions with androgen receptor (AR) and peroxisome proliferator-activated receptor gamma (PPARG) [21]. Additionally, a role for ZFHX4 in neuronal and muscle differentiation in mice has been suggested [22].

The CYB5R3 gene balances the NAD ^+^/NADH ratio within cells to maintain redox homeostasis. CYB5R3 has been associated with mitochondrial dysfunction in cancer [23], a process that has been shown to play a context-dependent role in the promotion of tumor growth [24].

Notably, in addition to the discovery of novel drug targets we also identify established targets by querying DrugBank [25]. Twelve of the most influential genes across the three time-points are targeted by FDA approved drugs including five oxidoreductases (GLRX2, CYB5R3, NDUFA7, AKR1B1, RRM2), two proteases (SELP, MME), a methyltransferase (ATIC), a membrane transporter (FOLR2), a carrier protein (NDUFAB1), a cytokine (A2M), and a complement component (C1R). Additionally, five of the most influential genes following 90 days of letrozole treatment are the targets of experimental compounds. These genes include two kinases (TK1, NME1), a protease (ADAMTS5), a receptor (EPCAM), and an acetylhydrolase (PAFAH1B3). The availability of drugs provides an opportunity to expedite biological validation of these targets with the potential to repurpose some of these drugs for the treatment of breast cancer.

## Discussion

Network biology provides a map of the molecular interactions that underlie biological function making it an intuitive and promising approach for the identification of potential drug targets based on their context-dependent relationships. Here we introduce influence networks as a novel approach to the identification of the most important or influential nodes within these networks as potential drug targets for cancer therapeutics. We apply this method to three coexpression networks generated from biopsies of ER ^+^ breast tumors during the course of neoadjuvant treatment with the drug letrozole to identify novel targets for the rational design of combination treatment strategies.

We have shown that few nodes are highly influential suggesting that nodes are more likely to be influenced than to be influential. Additionally, influence is a transitory nodal property changing as the network is rewired by letrozole treatment. In this way, the influence score reflects the context-dependent functions of genes. These results are consistent with a study of gene regulatory networks showing that nodes holding important positions within the network are transient as the interactions between transcription factors and target genes change in a condition dependent manner [26].

Measures of centrality are routinely used to determine the importance of nodes within biological networks [4]. One would expect degree centrality and influence to be correlated because the influence score is calculated as the sum of edge weights, so nodes of high degree will tend to have high influence. A correlation between influence and betweenness centrality is not as obvious but it can be explained by data showing that degree and betweenness are correlated [5]. However, these relationships are not absolute as hubs and bottlenecks are not necessarily highly influential and vice versa.

A study evaluating hubs and bottlenecks in protein networks concluded that degree is a better predictor of essentiality in undirected interaction networks while betweenness is a better predictor of essentiality in directed regulatory networks [5]. Influence networks convert undirected networks into directed networks where influence scores are a better predictor of essentiality than either degree or betweenness. The advantage of influence is that it incorporates information about second neighbors when determining the influence a given gene has on its partner. This captures asymmetric relationships between genes providing a better informed prediction of overall influence within the network.

A recent study to catalog knockout phenotypes in mice at the genome-wide level revealed that although there is a literature bias toward known genes [27], unknown genes are equally likely to underlie disease and to be essential [28]. Here we have identified three relatively unknown genes, TMEM14A, ZFHX4, and CYB5R3, as the most influential genes and thus the top candidates as potential drug targets across our networks. These targets are biologically relevant playing roles in cancer associated pathways including resisting apoptosis, invasion, differentiation and deregulating cellular energetics [29].

The full potential of molecularly targeted drugs lies in combination therapy. There are at least two ways to think about drug combinations for the treatment of cancer. One is synthetic lethality where redundant functions must be targeted together to cause cell death [30]. The other is induced-essential synthetic lethality where compensatory functions must be targeted together to cause cell death [30]. The distinction is that the compensatory functions are not activated until the cell has been rewired by a genetic mutation or environmental perturbation such as drug treatment. Here we incorporate the concept of induced-essentiality into drug target discovery by identifying targets that become influential only after the tumors have undergone 14 days or 90 days of letrozole treatment. This presents an opportunity to design combination therapies that sequentially add drugs to letrozole treatment as the tumors adapt. In this particular case, neoadjuvant letrozole is given for 90 days but recent trials have shown that patient tumors continue to respond to the drug for up to one year [31-34] suggesting that in this setting, sequential therapy is a practical strategy.

## Conclusion

As the molecular characterization of tumors has become routine, molecularly targeted drugs have become a mainstay for the treatment of cancer. The success of a targeted therapeutic depends not only on the identification of the most appropriate target but also on how it is combined with other targeted drugs. Here we have introduced influence networks as a method to prioritize genes as potential drugs targets. We have shown that as tumors adapt to treatment with the drug letrozole, these potential targets change, reflecting changes in the underlying function of genes in the presence of the drug. The results of our analysis have implications in the design of combination therapy treatment strategies that are applicable beyond letrozole and ER ^+^ breast cancer. In general, targeting influential genes as they become essential for tumor viability throughout the adaptation process presents an opportunity to enhance the effects of molecularly targeted drugs by staying a step ahead of resistance. Network-based metrics are an effective way to identify targets and their combinations based on the context-dependent roles they play within the system.

## Competing interests

The authors declare that they have no competing interests.

## Authors’ contributions

NP conceived of the study and performed the analyses. NP and JM designed the study and drafted the manuscript. Both authors read and approved the final manuscript.

## Declarations

The publication costs for this article were funded by JM.

## Supplementary Material

Additional file 1 Enrichment of essential genes among influential genes across thresholds. Click here for file

Additional file 2 Enrichment of essential genes among influential genes, hub genes, and bottleneck genes across thresholds. Click here for file

Additional file 3 Distributions of changes in influence scores among top candidates at each time-point relative to randomly sampled gene sets of equal size. Click here for file
