# Supplementary material for: Influence networks based on coexpression improve drug target discovery for the development of novel cancer therapeutics
Source: BMC Syst Biol. 2014 Feb 5;8:12. doi: 10.1186/1752-0509-8-12 (PMC3922430; doi:10.1186/1752-0509-8-12)
Supplement: Additional file 1 — Enrichment of essential genes among influential genes across thresholds. [file 1752-0509-8-12-S1.pdf]

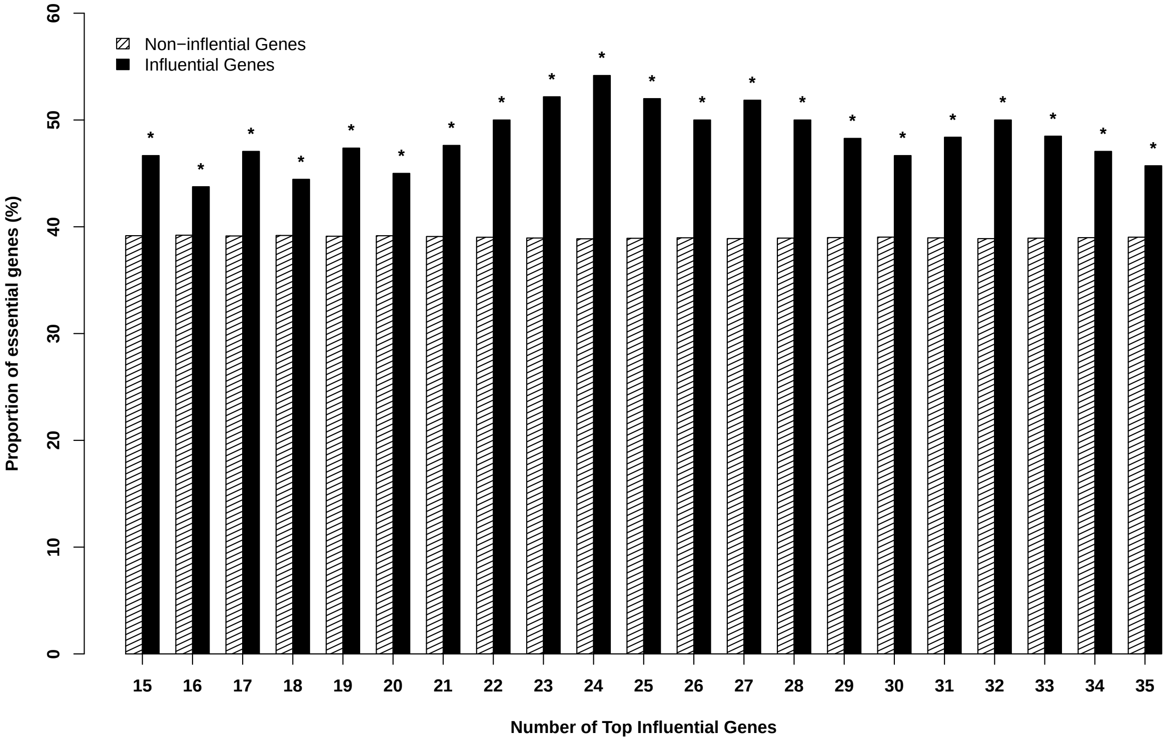

Supplemental Figure 1. Influential genes in a breast tumor coexpression network are enriched for essential genes at a range of thresholds. '*" indicates $p \leq .001$ calculated using the cumulative binomial distribution.
