# Supplementary material for: Influence networks based on coexpression improve drug target discovery for the development of novel cancer therapeutics
Source: BMC Syst Biol. 2014 Feb 5;8:12. doi: 10.1186/1752-0509-8-12 (PMC3922430; doi:10.1186/1752-0509-8-12)
Supplement: Additional file 2 — Enrichment of essential genes among influential genes, hub genes, and bottleneck genes across thresholds. [file 1752-0509-8-12-S2.pdf]

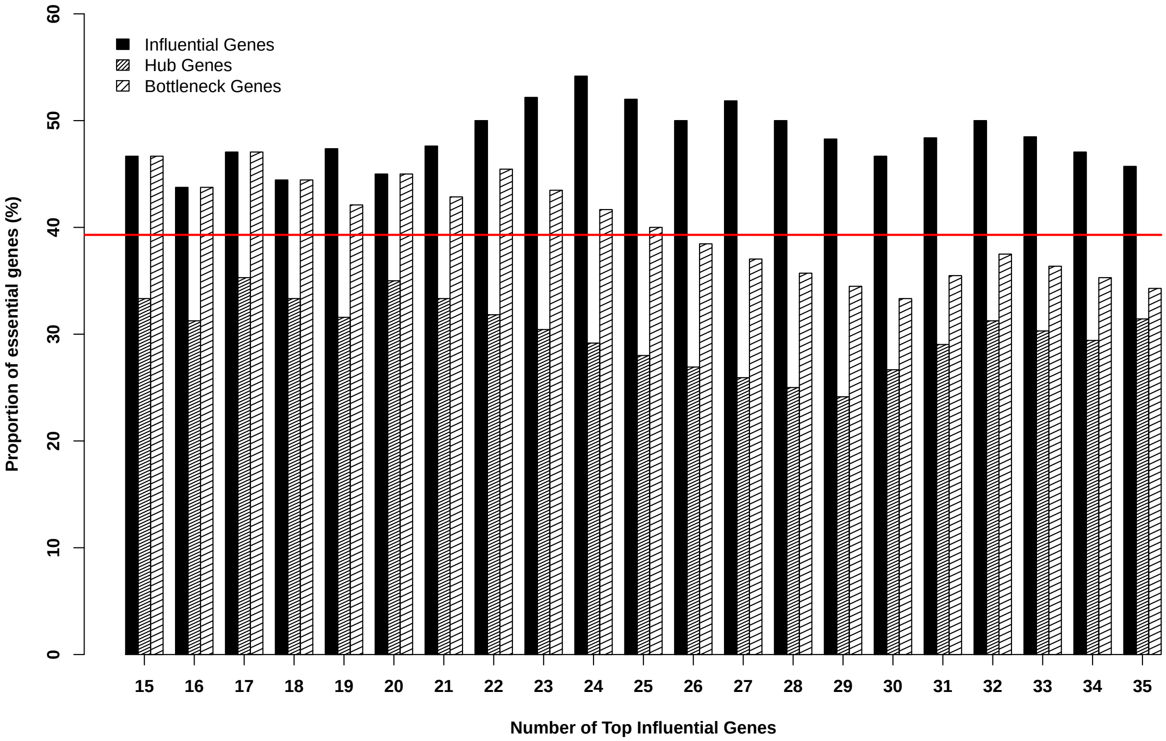

Supplemental Figure 2. Enrichment for essential genes among gene sets selected based on their influence score (influential genes), degree (hub genes), or betweenness centrality (bottleneck genes) in an untreated breast tumor coexpression network. The red line indicates the background enrichment observed for all genes in the network.
