# Supplementary material for: Influence networks based on coexpression improve drug target discovery for the development of novel cancer therapeutics
Source: BMC Syst Biol. 2014 Feb 5;8:12. doi: 10.1186/1752-0509-8-12 (PMC3922430; doi:10.1186/1752-0509-8-12)
Supplement: Additional file 3 — Distributions of changes in influence scores among top candidates at each time-point relative to randomly sampled gene sets of equal size. [file 1752-0509-8-12-S3.pdf]

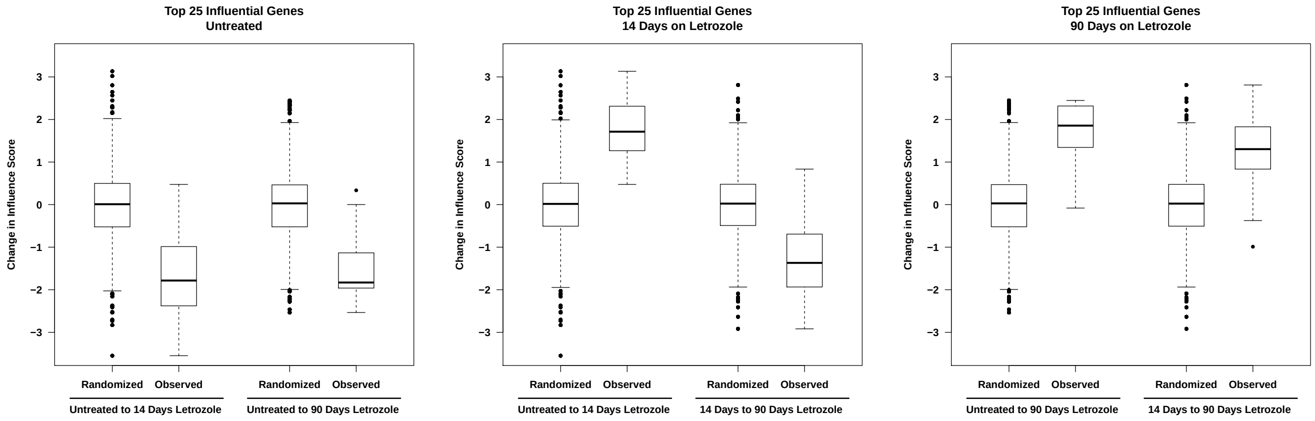

Supplemental Figure 3. The distributions of changes in influence score among the sets of most influential genes (Table 1) at each of three treatment time-points, are statistically significantly different from the distributions of changes in influence scores from equally sized gene sets chosen at random from the same networks. For each comparison, $p < .001$ as determined by Student's t-test.
